# Immobilization of alcohol dehydrogenase from *Saccharomyces cerevisiae* onto carboxymethyl dextran-coated magnetic nanoparticles: a novel route for biocatalyst improvement via epoxy activation

**DOI:** 10.1038/s41598-020-76463-x

**Published:** 2020-11-10

**Authors:** Katja Vasić, Željko Knez, Maja Leitgeb

**Affiliations:** 1 Faculty of Chemistry and Chemical Engineering, University of Maribor, Smetanova 17, 2000 Maribor, Slovenia; 2 Faculty of Medicine, University of Maribor, Taborska ulica 8, 2000 Maribor, Slovenia

**Keywords:** Biocatalysis, Immobilized enzymes

## Abstract

A novel method is described for the immobilization of alcohol dehydrogenase (ADH) from *Saccharomyces cerevisiae* onto carboxymethyl dextran (CMD) coated magnetic nanoparticles (CMD-MNPs) activated with epoxy groups, using epichlorohydrin (EClH). EClH was used as an activating agent to bind ADH molecules on the surface of CMD-MNPs. Optimal immobilization conditions (activating agent concentration, temperature, rotation speed, medium pH, immobilization time and enzyme concentration) were set to obtain the highest expressed activity of the immobilized enzyme. ADH that was immobilized onto epoxy-activated CMD-MNPs (ADH-CMD-MNPs) maintained 90% of the expressed activity. Thermal stability of ADH-CMD-MNPS after 24 h at 20 °C and 40 °C yielded 79% and 80% of initial activity, respectively, while soluble enzyme activity was only 19% at 20 °C and the enzyme was non-active at 40 °C. Expressed activity of ADH-CMD-MNPs after 21 days of storage at 4 °C was 75%. Kinetic parameters (*K*_M_, *v*_max_) of soluble and immobilized ADH were determined, resulting in 125 mM and 1.2 µmol/min for soluble ADH, and in 73 mM and 4.7 µmol/min for immobilized ADH.

## Introduction

In modern green chemistry, enzymes are valuable natural biocatalysts that possess some excellent properties, such as high activity, high selectivity and specificity, that allow even the most complex chemical reactions to be performed under mild experimental conditions^1–4^. Therefore, enzymes can find applications in various industrial processes and fields, such as biomedicine, nanotechnology, pharmaceuticals, biosensors, protein engineering, etcetera, and the main reason for such a wide application range is the enzyme stability. However, free enzymes are usually not stable in buffer solutions, but after immobilization (via covalent attachment, encapsulation, or simple adsorption), their stability can improve drastically^5–12^. Immobilization is a simple solution to many free enzyme related problems. Enzyme immobilization improves the controlling nature of the enzyme and eliminates many limitations by improving enzymes’ stability, selectivity or specificity, and even reduces enzyme inhibition^13,14^. Different supports may be applied using reversible methods, such as physical immobilization, and irreversible methods, such as covalent coupling^15–17^. Such immobilization protocols may even be applied to membrane technology for biocatalytic removal of pollutants^18^. Improving enzyme stability is one advantage of enzyme immobilization, since reusability of an enzyme is only possible when the activity of the enzyme can be maintained for several reaction cycles. However, not all immobilization methods are appropriate, nor do they all improve enzyme stability. If a less suitable immobilization method is used on the wrong support, this can even decrease the stability of an enzyme. Stability of an enzyme can also be improved by multipoint covalent attachment via short spacer arms that are attached on pre-existing rigid supports. For such immobilization, it is crucial to select an appropriate support, along with suitable reactive groups and immobilization conditions. Such supports have to present many reactive groups and offer low steric hindrances. Multipoint covalent attachment might be achieved using epoxides^19^, amino groups activated with glutaraldehyde^20^ or glyoxyl^21^ supports. While using non-porous supports, the specific area of such supports must be considered, since it depends on the particle size, which has to be in nano-scale in order to support reasonable enzyme loading capacity^14,22^. Enzymes immobilized onto non-porous materials can be attached to the support by multipoint in order to increase stability, as long as the enzyme is properly oriented on the surface of the support, while such multipoint attachments are not possible on a porous support. Nevertheless, there are some drawbacks to such immobilization onto an external surface, such as the non-protection of the enzyme from hydrophobic interfaces (in the form of gas bubbles). Also, proteolysis might occur, since enzyme molecules from one particle can interact with enzyme molecules from another particle. This can be improved by coating the support with polymers that prevent interactions and avoid enzyme inactivation. Another drawback involves diffusion limitations, which can decrease the expressed activity of enzymes, or even the stability of an enzyme^23,24^. Multipoint or multisubunit (in multimeric enzymes) immobilization can improve the rigidity of an enzyme and therefore improve its stability^1,4,25^. In addition, some immobilization strategies even provide the immobilization of an enzyme together with its purification in just one process step, without losing potential improvements to the enzyme. In industry, purified enzymes are expensive, which is the reason that only partially purified enzymes are used^14,25^. Also, enzyme selectivity and specificity can be improved by immobilization methods. However, since immobilization protocols may alter enzyme structure by changing the rigidity of specific areas or even changing the features of the enzyme, enzyme specificity and selectivity might change, as well. This change appears in the form of decreased or increased specificity and selectivity of the enzyme. In some cases, enzyme selectivity and specificity changes make an enzyme highly selective towards one specific enantioisomer or highly selective to another isomer. On the other hand, if free enzymes already have suitable properties, only a moderate immobilization should be applied to preserve such properties. In such cases, one-point covalent immobilization offers a better solution as an immobilization strategy^14,26,27^. Enzyme inhibition presents a problem in some reactions, since substrates, products or other compounds may cause a reduction in the reaction rate or even stop the reaction itself. Immobilization can be a solution to such problems, since enzymes that are being immobilized can increase their inhibitory constant (*K*_i_), which consequently affects their *K*_M_. With this cause, the enzyme inhibition is being reduced on the reaction course. Another effect is possible when the immobilization protocol blocks the enzyme inhibition site and therefore prevents enzyme inhibition^14^.

Iron oxide nanoparticles used as nano-carriers for enzyme immobilization represent a promising system in bionanomedicine, which has numerous clinical applications, such as cell targeting and labelling, targeted drug delivery, tissue repair and in biosensors^28–33^. When different magnetic nanoparticles (MNPs) were applied in a biosensor application, an amperometric biosensor was based on polydopamine-coated magnetite nanoparticles, which was used as a platform for the immobilization of glucose oxidase, alcohol oxidase and cholesterol oxidase, which were used to detect biomolecules^34^. Another study involves the synthesis of magnetite/lignin (Fe_3_O_4_/Lig) and magnetite/lignin/polydopamine (Fe_3_O_4_/Lig/PDA) materials, which were used for the immobilization of glucose oxidase, which can be applied in a biosensing application^35^. Magnetic nanoparticles (MNPs) have unique properties, such as superparamagnetism, large surface area and low toxicity^30,36–41^. However, they are inclined to aggregate, because of their strong magnetic dipole^42,43^. Since there are not enough active groups on the surface of nanoparticles, except for a few hydroxyl groups, their modification is in place. To overcome these restrictions and to make nanoparticles more biocompatible and biodegradable with low toxicity, various methods to functionalize the surface of MNPs using different organic polymers are employed to prevent degradation of iron oxide nanoparticles^44–48^. One such suitable organic polymer is dextran, which is a biocompatible, water-soluble, branched polysaccharide, made of many glucose molecules, comprising α-1, 6-glycosidic linkages and mostly used for health care products, including MRI and hyperthermia agents^49–51^. Dextran is produced by dextransucrases of various origins and has relatively high water solubility, high stability in both acidic and basic conditions and a large amount of hydroxylic groups. All these properties make dextran an excellent material for various kinds of chemical and physical cross-linking. There is an extensive review of dextran in biocatalysis, where it is described as an important and effective immobilization system^52^. A study by Shaterabadi et al. shows that the presence of dextran in a reaction medium results in significant suppression of the saturation magnetization of dextran-coated nanoparticles. Moreover, biocompatibility studies reveal that surface modification of nanoparticles by dextran coating decreases the cytotoxicity of uncoated nanoparticles and therefore improves their potential applications in biomedicine^53^. It has also been shown that dextran coating decreases iron nanoparticle-mediated cytotoxicity in pulmonary artery cells^54^, while another study shows that dextran-coated nanoparticles have no significant effect on synaptic vesicular functions^55^. There are also some acute toxicity and irritation studies, where the cytotoxicity of dextran-coated nanoparticles is attributed to the breakdown of the dextran shell exposing bare iron oxide nanoparticles, which interact with cellular systems^56^. Modified MNPs are usually further functionalized using different functionalization groups. The epoxy-activated group is a very active group, which can react with proteins, enzymes and nucleic acids, resulting in beneficial immobilization of biomolecules. Epoxy groups are also very stable at neutral pH values; therefore, different commercial supports can be prepared at a far position, where the enzyme has to be immobilized, which means epoxy-activated supports are very suitable biological systems to develop easy enzyme immobilization protocols^57,58^. EClH is a chlorinated epoxy compound and an epoxide, which is metabolized by binding to glutathione and by hydration via epoxide hydrolase with bifunctional alkylating activity. It is routinely used in the production of numerous synthetic materials, including epoxy, phenoxy and polyamide resins, cross-linked starch, surfactants and other pharmaceutical products^59–61^. However, immobilization onto epoxy-activated supports takes place in a two-step mechanism. In the first step, the adsorption of the enzyme is promoted, which means that the proposed immobilization on such support includes the usage of high ionic strength, which forces hydrophobic adsorption of the enzyme. In the second step, the enzyme that is adsorbed is covalently attached to the activated epoxy groups that are present on the surface of the support^57^. Two studies describe epoxy-functionalized nanoparticles, which were modified with silica, as a support for the immobilization of horse liver ADH to be a suitable carrier for immobilization of enzymes^62,63^, while another study shows immobilization of a lipase from porcine pancreas being covalently immobilized on the magnetic microspheres via the active epoxy groups^64^. There is also a study describing lipase immobilization onto epoxy-functionalized silica nanoparticles^65^ and another describing laccase immobilization onto epoxy-functionalized chitosan magnetic beads^66^. All reported studies show the importance and significance of epoxy-functionalized nanoparticles, suitable for enzyme immobilization.

In our study, we immobilized the enzyme ADH from *S. cerevisiae*, which is an important biological catalyst for oxidation of alcohols and reduction of aldehydes or ketones^67–71^. Therefore, ADH has many potential applications in various chemical industries, such as the pharmaceutical and food industry. It is important because of its yield and versatility in redox reactions^72–74^. Because of its enantioselectivity, it is an excellent catalyst in the pharmaceutical industry, since it can produce high-value enantiopure drugs^75,76^. ADH also has great application for production of various starting materials and intermediates, and synthesis of chiral compounds, but mainly for regeneration of the coenzymes NAD(P) and NAD(P)H. It has been applied in biosensor technology, as well^77–80^. However, ADH has low stability, which limits its use in industrial applications^81^, owing to its high sensitivity to temperature and pH. When applied under alkaline conditions, ADH’s tertiary structure distorts; when applied under acidic conditions, multimeric subunits of ADH dissociate, both of which cause the loss of ADH activity^82–86^. In order to stabilize and obtain the tertiary structure of such multimeric enzymes, an immobilization protocol of a multisubunit attachment to a very rigid support can be applied. Such an immobilization strategy requires that some of the crucial parameters be controlled (in general all parameters that play a role in improving the expressed activity of an immobilized enzyme, e.g. temperature, pH, immobilization time, enzyme concentration etc.). However, immobilizing under optimal conditions does not always guarantee fully stabilisation of the tertiary structure of multimeric enzymes, since the subunits might not be in the same place, making it impossible to perform stabilisation of an enzyme via the immobilisation protocol. In such a case, the enzyme will release the subunits into the reaction medium and become deactivated^87^.

There are studies describing immobilization of liver horse ADH inside porous supports^88^, while others show covalently immobilized ADH onto MNPs via a glutaraldehyde coupling reaction, where it managed to retain 49% of its original activity^89^. Later, there was a study describing immobilization of ADH developed by using surface functionalization of chitosan-coated MNPs, which retained 65% of its original activity^44^. More recent studies investigate immobilization of ADH on agarose activated with glyoxyl groups in the presence of acetyl cysteine, which results in 25% of expressed activity^85^. Anther research report describes immobilization of ADH onto silica nanoparticles functionalized with amino, epoxy and thiol groups, where a decrease of 60–70% in specific activities was observed^62^. Moreover, immobilization via covalent binding on crystalline Ni–Co nanoferrites, synthesized via sol–gel auto combustion techniques, was investigated, resulting in 40% expressed activity of immobilized ADH^90^. Immobilization of ADH onto polyaniline-coated silver nanoparticles, where it maintained 73% of the initial activity^91^, was also reported. However, there is a study describing immobilization of ADH onto epoxy-functionalized silica coated MNPs, where expressed activity of 92% was maintained at pH 8 and 30 °C with a 12-h immobilization time^63^, which proves the beneficial advantage of epoxy-functionalized nanoparticles for ADH immobilization. In comparison, our study proved better thermal stability of immobilized ADH, since it retained 80% of its initial activity after 24 h at 40 °C, while soluble ADH was inactivated under the same conditions.

**Figure 1:**
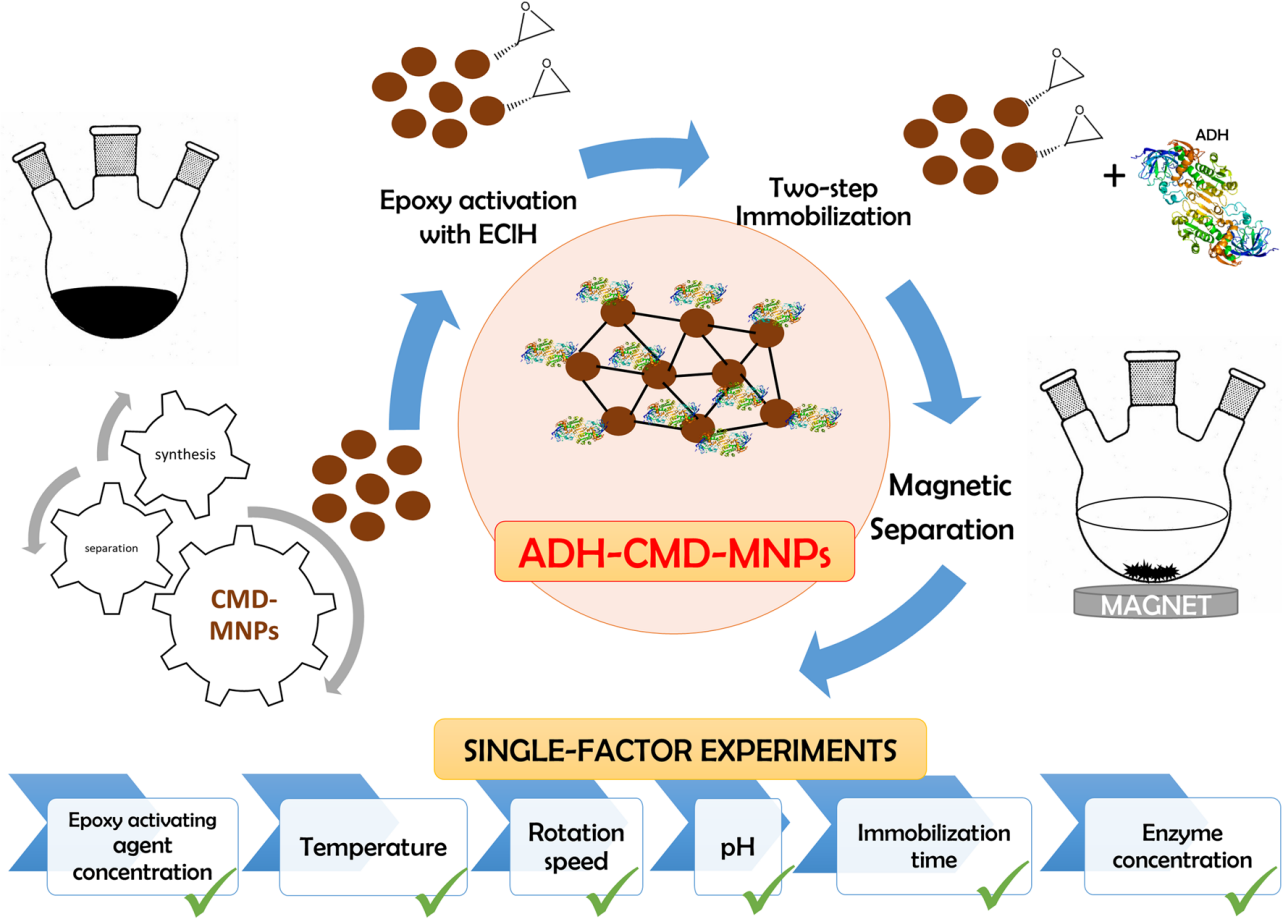
A schematic overall idea of our proposed manuscript describing immobilization of ADH onto epoxy activated CMD-MNPs via two-step mechanism; optimization of process parameters performed in single-factor experiments and the characterization of prepared biocatalyst (ADH-CMD-MNPs).

There are many research reports describing immobilization methods for binding different protein molecules to different dextran-coated MNPs^92–97^. There are also studies describing the use of CMD-MNPs in various applications^98–102^. However, there are only a few that describe the activity of ADH being affected by using different dextrans^103–105^, and none reporting about immobilization of ADH onto CMD-MNPs via epoxy activation, which makes our study a novelty. In this study, CMD-MNPs were prepared and later epoxy activated with the epoxide EClH as an activating agent, to be used as a carrier for immobilization of ADH. Thus, it is a novel method for immobilization of ADH onto CMD-MNPs via epoxy activation in a two-step mechanism. The overall idea of our study in presented in Fig. 1. The process factors (EClH concentration, temperature, rotation speed, medium pH, immobilization time and enzyme concentration) were optimized to obtain the highest expressed activity and immobilization yield of the immobilized ADH-CMD-MNPs. Characterization of the prepared nano-carrier CMD-MNPs and immobilization of ADH onto non-activated CMD-MNPs was published in our previous work^106^, while in this study, detailed characterization of immobilized ADH-CMD-MNPs was performed, while investigating its morphology and structure using FT-IR, TGA, SEM and DLS.

## Results and discussion

### Preparation and activation of ADH-CMD-MNPs

CMD-MNPs were synthesized by the co-precipitation method as described in our previously published article^106^. Subsequently, 20 mg of CMD-MNPs were activated with epoxy groups, using EClH, which was used as an agent for activating the ADH enzyme molecules and the CMD-MNPs. A specific volumetric ratio (% (v/v)) of EClH was used, and 0.1 M sodium phosphate buffer (pH 7.5) was added so that the final volume was 1 mL. Since EClH was incubated with CMD-MNPs and ADH, this resulted in the formation of an activated matrix of the enzyme and CMD-MNPs. ADH was immobilized via covalent binding between epoxy groups of CMD-MNPs and hydroxyl groups of ADH, which can also be observed from FT-IR spectra. It is well known that EClH can covalently bind amino (–NH_2_), sulfhydryl (–SH) and hydroxyl (–OH) moieties on proteins^107^.

### Immobilization of ADH-CMD-MNPs

#### Effects of process parameters on ADH immobilization

**Figure 2:**
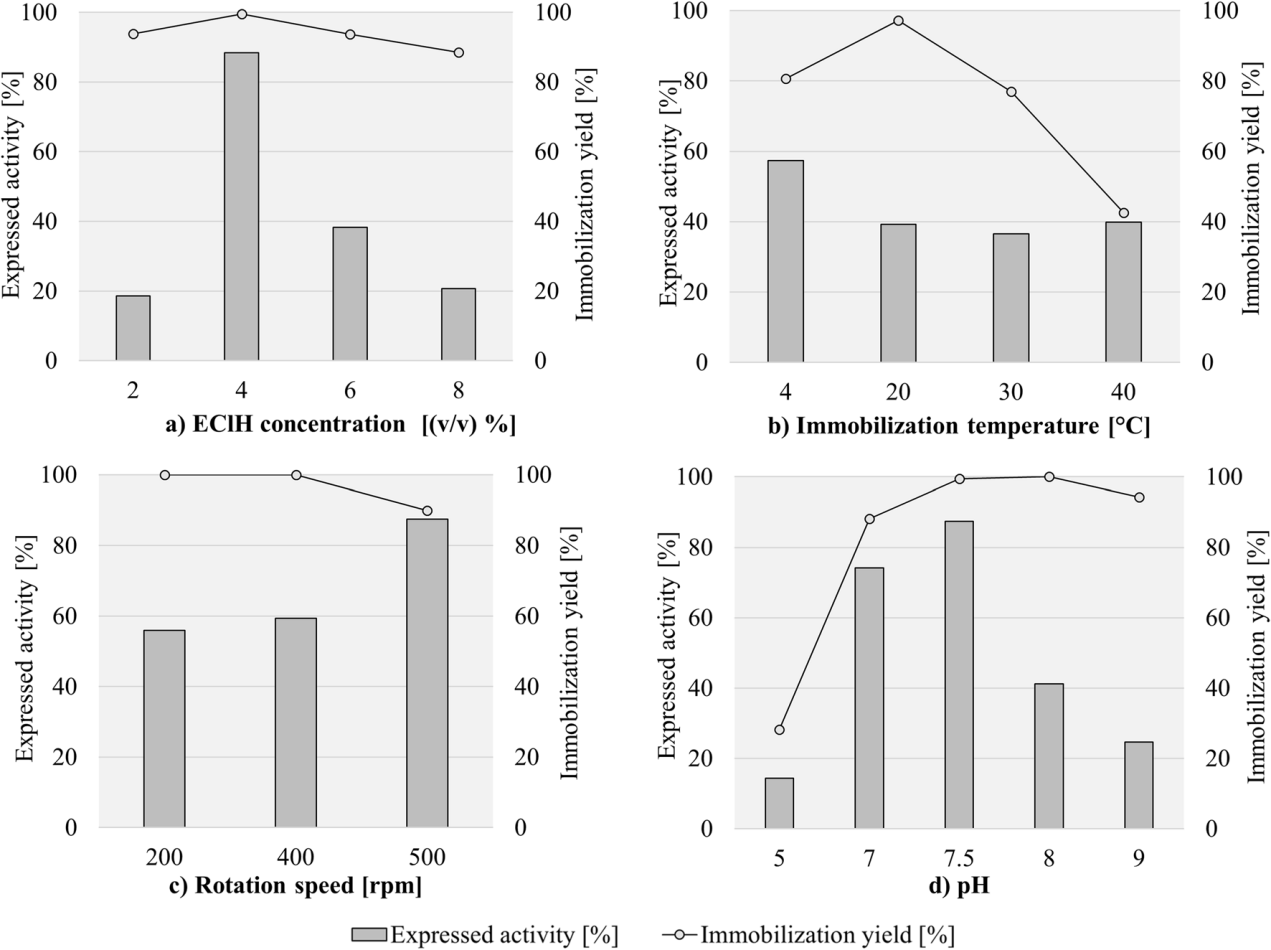
Expressed activity and immobilization yield of ADH-CMD-MNPs with the change in EClH concentration, immobilization temperature, rotation speed and pH value of immobilization medium. (Standard deviation for all samples was less than 2%; constant process conditions available in Table 2).

The concentration of EClH was optimized by performing immobilization of ADH on CMD-MNPs with different volumetric ratios of 0.5 M EClH as an activating agent, ranging from 2% (v/v) to 8% (v/v) with a final volume of 1 mL using a sodium phosphate buffer (pH 7.5). The activation was performed for 1 h at 300 rpm at 20 °C. After 1 h, the enzyme ADH (7.4 U) with additional sodium phosphate buffer (pH 7.5) in a volumetric ratio 1:9 (final volume 1 mL) was added to the activated CMD-MNPs, so that the final volume of the enzyme was 0.02 mg/mL. The immobilization was performed for 2 h at 400 rpm and 4 °C. Enzyme expressed activity was determined with the help of an enzymatic assay for ADH determination, using ethanol as a substrate, and immobilization yield was determined by estimating the protein concentration with the Bradford method in supernatants after immobilization and twice-repeated washing of the immobilized ADH-CMD-MNPs. As shown in Fig. 2a, when applying 2% (v/v) of EClH, the expressed activity was very low, only 19%. This could suggest that low activating agent concentration yields poor mechanism strength, easily leading to leaching of the enzyme from the carrier support. By increasing the concentration of the activation agent EClH to 4% (v/v), the expressed activity began to increase and proved to be the optimal volumetric concentration, since it retained 88% of the original enzyme activity, which corresponds to 6.5 U/mL. With increasing activating agent concentration, the number of free active groups on the surface of the carrier resulted in higher ADH loading, causing higher expressed activity of immobilized ADH. Also, immobilization yield was very high, resulting in 100%. In this case, immobilization yield goes hand-in-hand with the expressed activity of ADH. With further increasing the concentration of EClH, expressed activity and enzyme immobilization yield began to decrease. At 6% (v/v) of EClH, expressed activity decreased drastically to 38% and even more when applying 8% (v/v) of EClH, which resulted in 21% activity. This suggests that the limit of activation had been reached. Too high a concentration of the activating agent can result in enzyme deactivation, as well as in loss of activity, because excess concentrations of the activating agent result in blocking of the enzyme active groups^108^. Immobilization yield was 89% for 8% (v/v), 94% for 6% (v/v) and 94% for 2% (v/v) of used EClH. Immobilization of ADH onto non-activated CMD-MNPs was described in our previously published article^106^, where no activation of support with EClH was performed. Immobilization was performed at two different temperatures, 4 °C and 20 °C, and resulted in only 26% and 15% of expressed activity of immobilized ADH, respectively. This study shows that activating the support with epoxy groups significantly improves the expressed activity of immobilized ADH. The 62% increase in expressed activity of immobilized ADH resulted in 88% of expressed activity of immobilized ADH onto epoxy-activated CMD-MNPs.

The temperature at which the immobilization is performed is an important factor affecting expressed enzyme activity and immobilization yield, as well as the operational nature of the immobilized enzyme. The immobilization protocol was performed using an optimized volumetric ratio of EClH for activation of CMD3-MNPs from the first optimization step, which was 4% (v/v) with a 0.1 M sodium phosphate buffer (pH 7.5). Immobilization was performed for 2 h at 400 rpm with an enzyme concentration of 0.02 mg/mL, varying the immobilization temperature, which was 4 °C, 20 °C, 30 °C and 40 °C. The effect of immobilization temperature is presented in Fig. 2b, where it can be observed that the highest expressed activity was maintained at 4 °C, resulting in 57%. Immobilization yield reached 81%. At a lower immobilization temperature, the enzyme acts in a more stable manner and yields high immobilization yield, which can be used in further optimization protocols; it also offers broad possibilities in various applications. Immobilization performed at 20 °C, 30 °C and 40 °C resulted in a decrease in expressed activity (39%, 37% and 40%, respectively), with immobilization yield figures of 97%, 77% and 43%, respectively.

Another important factor influencing the expressed activity of immobilized enzymes is rotation speed, where high rotation speed preferably results in higher expressed activity, since the bond between epoxy-activated MNPs and the enzyme is stronger and therefore more successful, as it allows more contact between the enzyme and the support^109^. When optimizing the rotation speed of immobilization, the same conditions were used as those for activation of CMD-MNPs, which were 4% (v/v) of EClH with a sodium phosphate buffer (pH 7.5) and a duration of 1 h at 300 rpm and 20 °C. When performing immobilization for 2 h, an enzyme concentration of 0.02 mg/mL was applied and the optimal temperature from the previous step, which was 4 °C at different rotation speeds (200 rpm, 400 rpm and 500 rpm), which were studied in order to obtain the highest expressed activity of immobilized ADH. As can be seen from our results in Fig. 2c, when applying 200 rpm to the immobilization process, only 56% of expressed activity was maintained. After doubling the rotation speed to 400 rpm, the expressed activity slightly increased to 59%. With a further increase in rotation speed to 500 rpm, the highest expressed activity was maintained, resulting in 88%, but at these conditions, a drop in immobilization yield was observed. Immobilization yield was 100% for 200 rpm and 400 rpm, but a slight decrease is observed at 500 rpm, resulting in still value (90%). The reason for the slight drop in immobilization yield can be the stronger shear forces occurring in the immobilization process at higher rotation speeds^110,111^, which on the other hand, did not affect the enzyme’s expressed activity.

Enzyme action and the properties of the immobilized enzyme are strongly influenced by the surrounding micro-environment in which the immobilization process is performed. An important parameter in this case is the pH of the immobilization medium. The pH of the solution also dictates the amount of enzyme loaded to the surface of the carrier by altering the electronic properties of the enzyme and the carrier. Each enzyme has its own optimal pH, as does ADH from *S. cerevisiae*, for which it is in the pH range 5–9^112^. Thus, ADH was immobilized under different pH conditions in the range from 5 to 9, using all previously optimized conditions for activation of CMD-MNPs and immobilization of the enzyme ADH, which were 4% (v/v) of EClH, 300 rpm and 20 °C. The immobilization protocol lasted for 2 h at 4 °C and 500 rpm with 0.02 mg/mL enzyme concentration, varying the pH of the immobilization medium. As seen from the results in Fig. 2d, the highest expressed activity was observed in pH medium 7.5 (87%). Expressed activity of immobilized ADH began to decrease the closer it approached to a more alkaline or more acidic micro-environment. Protein denaturation may occur under conditions of excess alkali or acid^113^; that is why it is important to select an appropriate pH value for the immobilization procedure. The lowest expressed activity was reached at the most acidic pH value (pH 5), 14% with an immobilization yield of 28%. In addition, the most alkaline pH value (pH 9) also resulted in a significant loss of enzyme activity to only 25%, with an immobilization yield of 94%. When a multimeric enzyme such as ADH is applied under acidic conditions, its multimeric subunits dissociate. Furthermore, when applied under alkaline conditions, its tertiary structure distorts, both of which cause a loss of its activity^87^.

Nevertheless, when immobilizing such an enzyme on an epoxy-activated support, the immobilization involves a two-step mechanism^57^. First, the functional groups of the enzyme ADH are adsorbed on the surface of an epoxy-activated support, such as epoxy-activated CMD-MNPs. Second, the enzyme ADH that is adsorbed becomes covalently attached to the epoxy-activated support of CMD-MNPs.

**Figure 3:**
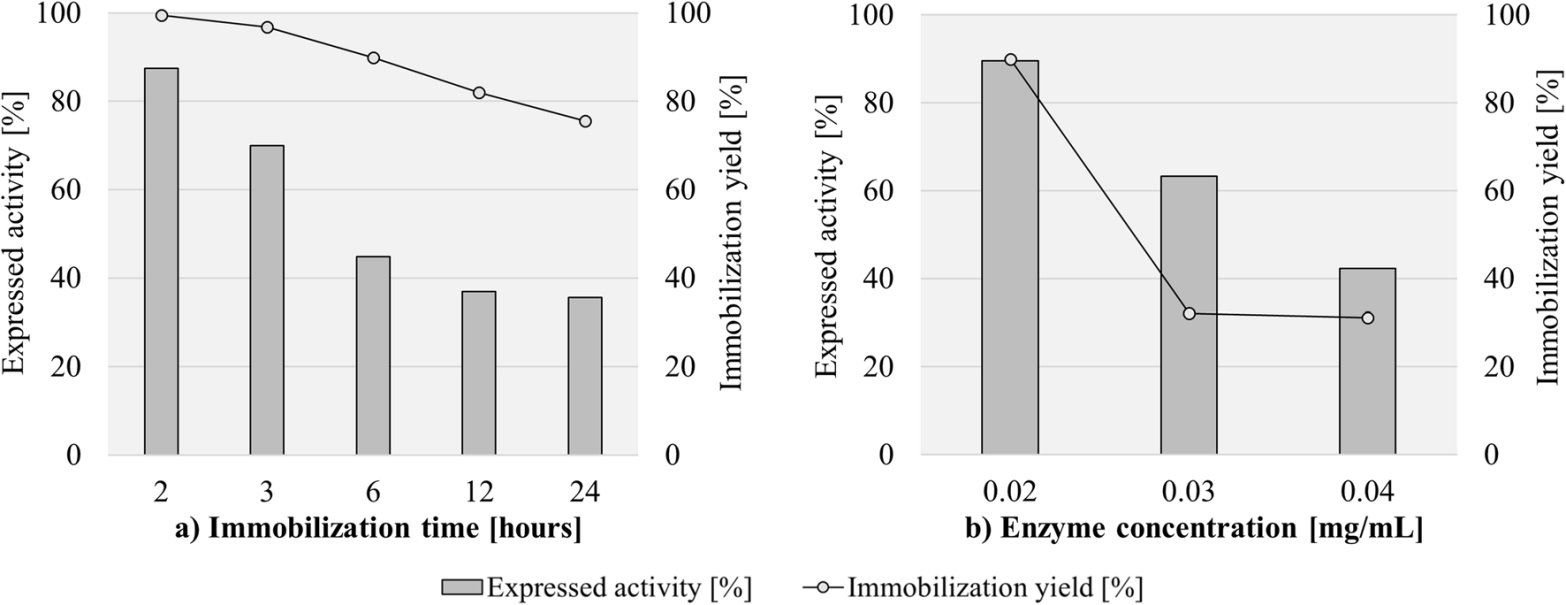
Expressed activity and immobilization yield of ADH-CMD-MNPs at different immobilization times and different enzyme concentrations. (Standard deviation for all samples was less than 2%; constant process conditions available in Table 2).

The influence of immobilization time is presented in Fig. 3a. At first, immobilization time expanded over the period of 24 h; during optimization the time shortened gradually to 2 h, while using all optimized conditions from previous steps for activation of CMD-MNPs, using 4% (v/v) of EClH for 1 h at 20 °C and 300 rpm, and all conditions for immobilization, which was performed at 4 °C in pH 7.5 at 500 rpm with 0.02 mg/mL of ADH, varying the time of immobilization. At the longer immobilization time of 24 h, the expressed activity of immobilized ADH-CMD-MNPs maintained only 36%, with 76% of immobilization yield. When performing the immobilization for only 12 h, the expressed activity resulted in a slight increase, exhibiting 37% of expressed activity and 96% of immobilization yield. After splitting the duration of immobilization in half again, the expressed activity increased a bit further, resulting in 45% after 6 h, with 90% of immobilization yield. When the immobilization time was only 3 h, the expressed activity significantly increased and exhibited 70%, with 97% of immobilization yield. After another cut in immobilization time, the highest expressed activity of immobilized ADH-CMD-MNPs was maintained after just 2 h, resulting in 87%, with 100% of immobilization yield. The results indicate that the immobilization time has an important effect on the expressed activity of immobilized ADH-CMD-MNPs and that the optimal immobilization lasts for 2 h, which is sufficient time to bind the enzyme ADH effectively to the CMD-MNP surface with its highest possible activity. Since each enzyme has its own specificity, the immobilization time is of high importance. When the carrier is saturated with the enzyme on its surface and all active sites on the enzyme are occupied, the immobilization process of the enzyme onto the carrier stops. This can be exhibited in the form of lower expressed activity of the immobilized ADH.

It would be expected that an increase in ADH concentration would yield a more active product. On the contrary, with an increase in enzyme concentration to 0.03 mg/mL and 0.04 mg/mL, the expressed activity of immobilized ADH-CMD-MNPs decreased gradually, while using all optimized conditions from previous steps, as for activation, (4% (v/v) EClH for 1 h at 300 rom and 20 °C) and for immobilization protocols (2 h at 4 °C, pH 7.5 and 500 rpm). When the ADH concentration was increased from 0.02 mg/mL to 0.03 mg/mL, the expressed activity decreased from 90 to 63%, and the immobilization yield decreased from 90 to 32%. With a further increase of ADH concentration to 0.04 mg/mL, the expressed activity decreased even more, to 42%, and the immobilization yield decreased slightly more, to 31%. These results can be observed in Fig. 3b. However, the decrease in expressed activity with increasing enzyme concentration can be attributed to the oversaturation of the enzyme over the support surface. Moreover, high enzyme concentration clustered on the surface of the support can reduce enzyme activity, owing to limited active site accessibility and denaturation of the enzyme^114–117^. Furthermore, the immobilization protocol might distort the enzyme, when there are multi-interactions between the enzyme and the support. In addition, the active center of the enzyme might become blocked by the immobilization protocol and promote diffusion problems. Such problems may lead to precipitation or aggregation of a soluble enzyme in the anhydrous medium. One solution to this problem is the preparation of a flexible and hydrophilic spacer arm that is long and flexible, which is similar to the soluble enzyme and has no ability to precipitate or aggregate. Also, immobilization might cause new interactions between the enzyme and the support, which could alter the conformation of the enzyme and its catalytic activity^14^.

While most research describes the improvement in ADH’s activity after immobilization via investigation of temperature and pH effect, our study investigates the effect of six different process parameters on the expressed activity of ADH. Temperature and pH of the immobilization medium, along with rotation speed, activating agent concentration, immobilization time and enzyme concentration were investigated, as well. Shakir et al. reported about ADH immobilization on nanocrystalline Ni–Co ferrites used as magnetic support, where expressed activity of the immobilized ADH was 70% at 45 °C and pH 8^90^. Furthermore, ADH immobilized onto magnetic nanoparticles exhibited the highest expressed activity of 49% at 30 °C and pH 6.8 in research by Li et al.^89^. Another study by Li et al. describes the highest obtained expressed activity of 65% at pH 7.4 and 30 °C, where ADH was immobilized onto chitosan-coated MNPs^44^. Alam et al. investigated ADH immobilization on polyaniline-coated Ag nanoparticles, where the highest expressed activity of immobilized ADH was 73% at pH 8 and 40 °C^91^. Shinde et al. studied immobilization of ADH onto polyvinyl alcohol fibrous carriers, where immobilized ADH retained 60% of its original activity^84^. Research by Jiang et al. reports 92% expressed activity of immobilized ADH onto epoxy-functionalized silica MNPs at 30 °C and pH 8 after 12 h of immobilization^63^. All these reports show moderately high expressed activity of immobilized ADH on a range of carriers, but CMD-MNPs were not used in any of these studies. Our study presents the use of CMD-MNPs, which were epoxy-activated, as were the silica MNPs from the Jiang study^63^, where we also managed to obtain very high expressed activity of immobilized ADH-CMD-MNPs, a result of 90%. This is an important contribution to the improvement of the ADH immobilization process.

### Properties of immobilized ADH-CMD-MNPs

#### Thermal stability of soluble ADH and immobilized ADH-CMD-MNPs

**Figure 4:**
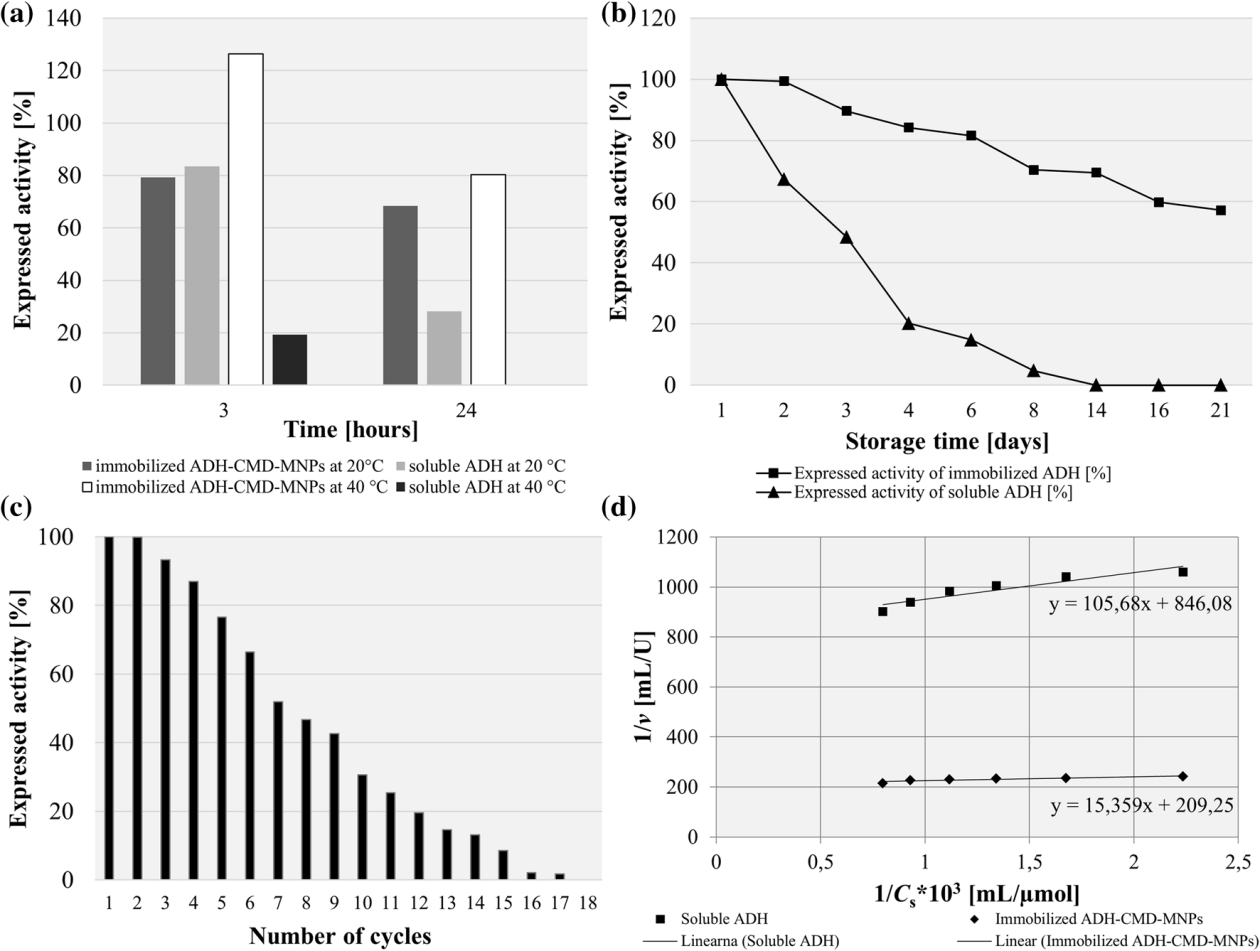
Thermal stability at 20 °C and 40 °C after 3 and 24 h (**a**), storage stability at 4 °C after 21 days (**b**) of immobilized ADH-CMD-MNPs and soluble ADH, reusability of immobilized ADH-CMD-MNPs after 18 consecutive cycles (**c**) and Lineweaver–Burk double reciprocal plot for soluble ADH and immobilized ADH-CMD-MNPs; substrate ethanol concentration was varied between 0.3 and 0.8 M for both soluble and immobilized ADH-CMD-MNPs (**d**). (Standard deviation for all samples was less than 2%).

As is well known, soluble ADH is very unstable, although it is highly active^85^. Increasing the thermal stability of ADH by immobilization is very important to provide a wider spectrum of use for different applications. Thermal stability of immobilized ADH-CMD-MNPs was studied at 20 °C and 40 °C after 3 h and 24 h of incubation, compared to soluble ADH treated under the same conditions. After 3 h of incubation at 20 °C, expressed activity of immobilized ADH-CMD-MNPs was 79%, while the activity of soluble ADH was 83%. However, with prolongation of incubation time to 24 h, expressed activity of immobilized ADH-CMD-MNPs decreased to 68%, while soluble ADH exhibited only 28% of expressed activity. When incubating immobilized ADH-CMD-MNPs at 40 °C, its expressed activity after 3 h of incubation resulted in 126%, and when the incubation time was prolonged to 24 h, although the expressed activity of immobilized ADH-CMD-MNPs dropped to 80%, the enzyme was still found to be very stable. In the comparison, soluble ADH had very low enzyme activity at 40 °C after 3 h (19%) and was, as expected, inactive after 24 h of incubation at 40 °C. In addition, we investigated the expressed activity of soluble ADH after 5 h at 40 °C, and the enzyme was also found to be inactive. These results are presented in Fig. 4a and suggest that our immobilization protocol, using epoxy-activated CMD-MNPs as support for ADH managed to improve enzyme stability even after 24 h of incubation at 20 °C and 40 °C. The change in expressed activity of immobilized ADH-CMD-MNPs during incubation at 40 °C could also be attributed to a combination of a certain temperature and incubation time with epoxy-activated CMD-MNPs, which can cause a change in the catalytic capability of the enzyme^114^.

Highly improved thermal stabilization of the immobilized ADH offers the opportunity to use this enzyme in a broader range of applications. A thermal study by Bolivar et al. reported less than 30% of expressed activity of immobilized ADH after 24 h at 63 °C^88^, while Li et al. reported 80% of expressed activity of immobilized ADH after just 1 h at 50 °C^89^. Moreover, Shinde et al. investigated the thermal stability of immobilized ADH after just 2 h at 60 °C and 80 °C, which resulted in 80% and 60% of expressed activity of immobilized ADH, respectively^84^. Jiang et al. reported 89% of expressed activity of immobilized ADH after 3 h at 50 °C^63^. Research studies by Shakir and Alam both report 5-h investigations of thermal stability, where Shakir et al. reported 45% of expressed activity of immobilized ADH onto CoFe_2_O_4_ MNPs and 66% of expressed activity of immobilized ADH onto NiFe_2_O_4_ MNPs, both of which were investigated at 60 °C^90^. However, Alam et al. reported 71% of expressed activity of immobilized ADH at 50 °C^91^. In comparison, our study yielded better thermal stability of immobilized ADH, since it retained 80% of its initial activity after 24 h at 40 °C, while soluble ADH was inactivated under the same conditions.

#### Storage stability of solube ADH and immobilized ADH-CMD-MNPs

Storage stability of immobilized ADH is another important factor for its application. To investigate storage stability, immobilized ADH-CMD-MNPs and soluble ADH were incubated at 4 °C for 21 days, and expressed activity was determined at certain time intervals, compared to the expressed activity of ADH-CMD-MNPs on day 1. The results are shown in Fig. 4b. After being placed at 4 °C, immobilized ADH-CMD-MNPs managed to maintain 82% of its expressed activity after 6 days, while soluble ADH lost more than 80% of its initial activity and maintained only 15% after 6 days. On day 8, expressed activity of immobilized ADH-CMD-MNPs decreased to 71%, while soluble ADH showed only 5%. After 14 days, the expressed activity of immobilized ADH-CMD-MNPs further decreased to 70%, while soluble ADH completely deactivated and lost all of its activity. After 16 days, immobilized ADH-CMD-MNPs still maintained 60% of the expressed activity. The activity drop was found to be linearly dependent on storage time, and immobilized ADH-CMD-MNPs maintained 60% of the expressed activity after 21 days at 4 °C. This confirms the very strong enzyme stabilization, since after 21 days of storage, the half-life of the immobilized enzyme was still not reached.

#### Reusability and half-life

Reusability is an important parameter that evaluates the yield of the immobilized enzyme and its usage on a large scale. Figure 4c shows the expressed activity of immobilized ADH used for 18 consecutive cycles, where it can be observed that immobilized ADH exhibited activity after 17 cycles and maintained more than 60% of initial activity even after 6 consecutive cycles. Half-life of immobilized ADH was reached after 8 cycles. This indicates that the immobilized ADH has good recovery, with minimum leakage and good durability.

### Kinetic constant determination of soluble and immobilized ADH

In order to determine the *K*_M_ of soluble and immobilized ADH, the peak area of the product for a series of concentrations of ethanol was determined (at 0.3 M, 0.4 M, 0.5 M, 0.6 M, 0.7 M to 0.8 M). According to Eq. (6), the linear regression obtained for soluble ADH was y = 105.68x + 846.08, and the *K*_M_ value of soluble ADH was obtained from the slope and maximum reaction velocity, which was calculated and resulted in 125 mM, while *v*_max_ resulted in 1.2 µmol/min. The linear regression for immobilized ADH-CMD-MNPs was y = 15.359x + 209.25, and the *K*_M_ value of immobilized ADH was obtained from the slope and maximum reaction velocity, as well, which was calculated, resulting in 73 mM, while *v*_max_ resulted in 4.7 µmol/min (Fig. 4d). The lower *K*_M_ value of immobilized ADH indicates its higher affinity towards the substrate. The reason may lie in the smaller size of the CMD-MNPs prepared for ADH immobilization, which causes a decrease in steric hindrance of the nanoparticles, thus increasing the accessibility of active enzyme sites to the substrate^63^. With that, the lower *K*_M_ value of our immobilized ADH indicates that our immobilization protocol improved ADH’s enzymatic performance, while improving its stability and potential for reusability. The lower *K*_M_ confirms the improvement in immobilized ADH through our epoxy-activated immobilization protocol onto CMD-MNPs.

The comparison of enzyme properties using different immobilization protocols for ADH is shown in Table 1. ADH immobilized onto polyaniline-coated Ag nanoparticles and magnetic crystalline Ni–Co nanoferrites gave higher *K*_M_ values of 205.3 mM and 237 mM, respectively. Moreover, the expressed activity of immobilized ADH was only 70% and 73%, respectively^90,91^. Other immobilization protocols which gave lower *K*_M_ values used different chitosan- or silica-coated nanoparticles and resulted in 37.77 mM and 31.32 mM, respectively^63,89^. However, Li et al. managed to obtain only 49% expressed activity of immobilized ADH, which was lower than in our study^89^. As in our study, Jiang et al. also used epoxy-functionalized MNPs, and the *K*_M_ value was low as well, resulting in 32.32 mM. The expressed activity of immobilized ADH was high, as in our study, resulting in 92%^63^. In addition, the investigation by Jiang et al. proves the good thermal stability of immobilized ADH, which resulted in 89% of expressed activity after just 3 h at 50 °C^63^, while our thermal stability study even improved those results, resulting in 126% of expressed activity after 3 h and in 80% of expressed activity after 24 h at 40 °C. Our study and the study by Jiang et al. prove the beneficial properties of MNPs being functionalized with highly active epoxy groups, which results in high expressed activity of immobilized ADH and in low *K*_M_ values. The increase or decrease in *K*_M_ values indicate that immobilized ADH has an apparent lower or higher affinity towards the substrate; in our case, lower *K*_M_ value of immobilized ADH compared to soluble ADH shows an improvement brought by our immobilization protocol. Therefore, the immobilized ADH shows improved biocatalytic properties in possible applications. Compared to other carriers, epoxy-functionalized nanoparticles seem to be more suitable for immobilization of ADH, since epoxy groups of functionalized MNPs bind successfully with functional groups of ADH, and can therefore considerably improve ADH expressed activity after immobilization protocols.

**Table 1:** Comparison of enzyme properties using different immobilization protocols for ADH.

Carrier	NP size	Expressed activity (%)	*K*_M_ (mM)	References
Glyoxyl-agarose	N.D.	50	N.D.	Bolivar et al.^88^
Magnetic chitosan Fe_3_O_4_ nanoparticles via glutaraldehyde coupling	25 nm	49	37.77	Li et al.^89^
Fe_3_O_4_ bound with chitosan alpha ketoglutaric acid	~ 26 nm	65	N.D.	Li. et al.^44^
Silica nanoparticles (functionalized with epoxy groups)	~ 50 nm	76	N.D.	Petkova et al.^62^
Agarose activated with glyoxyl groups in the presence of acetyl cysteine	N.D.	25	N.D.	Bolivar et al.^85^
Magnetic crystalline Ni–Co nanoferrites	20–30 nm	70	237	Shakir et al.^90^
Polyaniline coated AgNPs	~ 30 nm	73	205.03	Alam et al.^91^
Epoxy-functionalized silica-coated Fe_3_O_4_ nanoparticles	~ 24 nm	92	31.32	Jiang et al.^63^
Polyvinyl alcohol (PVA) fibrous carrier	N.D.	60	N.D.	Shinde et al.^84^
Epoxy-activated CMD-coated MNPs	57–78 nm	90	73	This work

*N.D.* not determined.

### Characterization of immobilized ADH-CMD-MNPs

After process of immobilizing ADH onto synthesized CMD-MNPs, characterization of the immobilized ADH-CMD-MNPs was performed, to obtain the structural properties of our CMD-MNP carrier with the immobilized enzyme. The presence of immobilized ADH onto CMD-MNPs was confirmed by FT-IR and TGA analysis, while the size and morphology of immobilized ADH-CMD-MNPs were investigated using SEM and DLS techniques.

#### FT-IR spectroscopic studies

**Figure 5:**
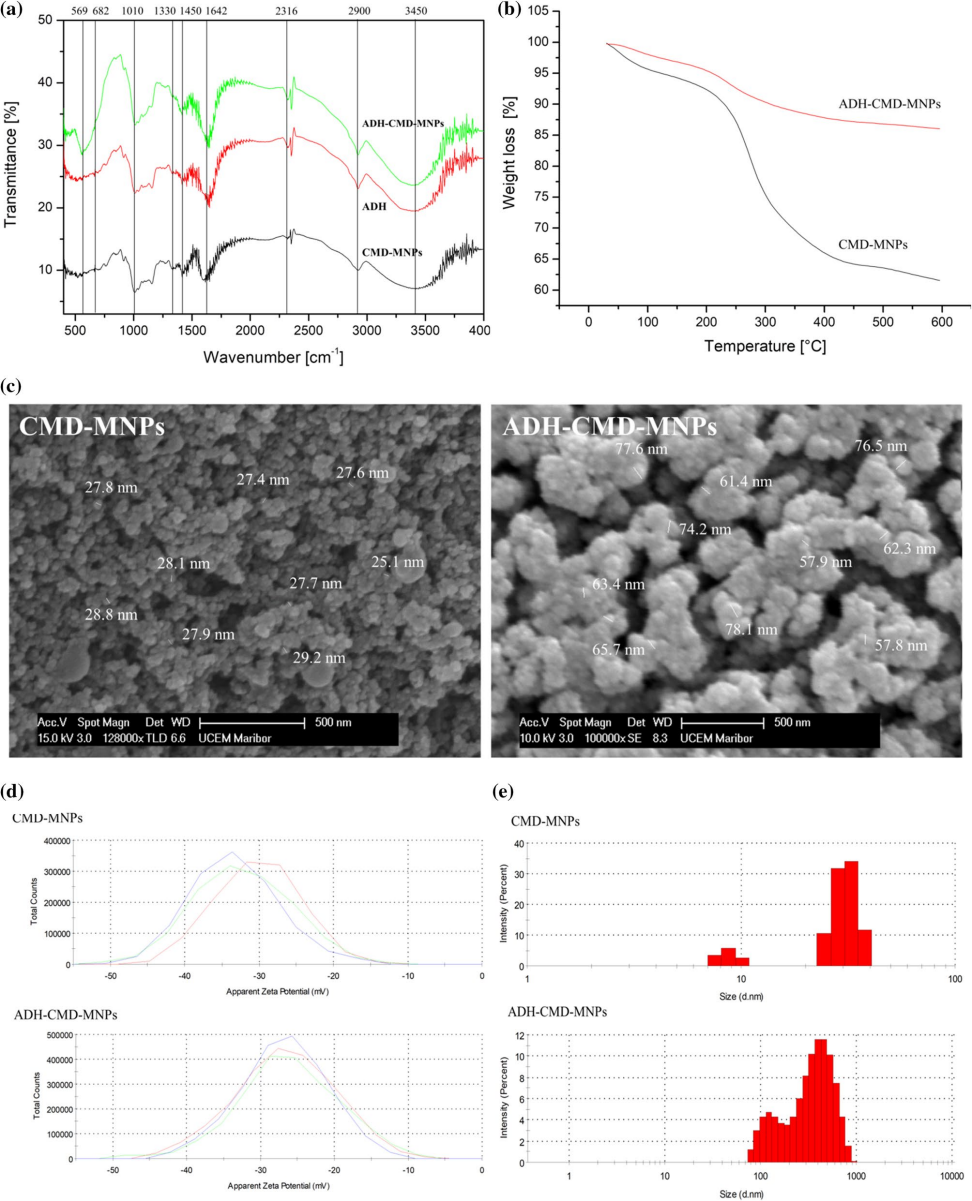
FT-IR spectra of soluble ADH, CMD-MNPs and ADH-CMD-MNPs (**a**), TGA curves (**b**), SEM images (**c**), zeta potential (**d**) and size distribution by intensity (**e**) of CMD-MNPs and ADH-CMD-MNPs; values are presented in a graph of three independent measurements for each sample.

The FT-IR spectra of CMD-MNPs, ADH-CMD-MNPs and soluble ADH presented in Fig. 5a were studied. The absorption peaks at 569 cm^−1^ and 682 cm^−1^ belong to the stretching vibration modes of the Fe–O bond, which indicate that the MNPs were successfully synthesized. Characteristic adsorption peaks are shown at 2900 cm^−1^ and 1375–1460 cm^−1^, and these belong to CMD νC–H and δC–H vibrational modes, while characteristic peak at 917 cm^−1^ belongs to α-glucopyranose ring^98^. The absorption peaks at 1010 cm^−1^ and 1250 cm^−1^ correspond to CMD νC–O vibrations^118^, while the broad absorption peak at 3408 cm^-1^ corresponds to the characteristic νO–H stretching and δO–H deformation modes of CMD hydroxyl groups, while CMD carboxyl groups are represented by the absorption peak at 1642–1730 cm^−1^. The ADH enzyme complex in ADH-CMD-MNPs shows characteristic absorption peaks at 1330 cm^−1^, 1641 cm^−1^ and 1450 cm^−1^, which can also be observed in the soluble ADH spectra, exhibiting characteristic frequencies of the ADH complex at 1641 cm^−1^ and 1388 cm^−1^. Furthermore, the symmetric ring stretching frequency of the epoxy ring around 831, 921, and 1249 cm^−1^ was presented^119^, indicating the existence of the epoxy group on epoxy-activated CMD-MNPs.

#### Thermogravimetric analysis

The TGA of CMD-MNPs and ADH-CMD-MNPs is presented in Fig. 5b. TGA represents the change in mass, which is dependent on increasing temperature. The TGA curve of CMD-MNPs shows that initial weight loss pertains to the temperature range of 70–150 °C, which is due to the loss of physically adsorbed water. The second weight loss pertains to the temperature range of 150–300 °C. This is the temperature required to induce thermal degradation of polymers, which corresponds to decomposition of CMD. We can observe almost 40% mass loss of CMD-MNPs. The epoxy-activated ADH-CMD-MNPs begin degradation at a similar range of 70–150 °C, which also corresponds to the initial loss of adsorbed water. It can be observed that for epoxy-activated ADH-CMD-MNPs, degradation begins in the temperature range from 400–450 °C, which corresponds to the degradation temperature of epoxy-activated materials, which is around 440 °C. Our TGA study shows that at 600 °C, there is approx. 20% of mass loss detected, which indicates that immobilization of ADH via epoxy activation increases the thermal stability of ADH-CMD-MNPs^120^, as non-immobilized ADH would formulate to ash after 100 °C and CMD-MNPs lose 40% of its initial mass above 400 °C. This confirms the epoxy-activated structure of ADH-CMD-MNPs^121,122^.

#### SEM micrographs of CMD-MNPs and ADH-CMD-MNPs

The morphology of epoxy-activated CMD-MNPs with immobilized ADH was investigated by SEM. Figure 5c shows SEM images of CMD-MNPs without bound ADH (a) and epoxy activated CMD-MNPs with immobilized ADH. It is clear that CMD-MNPs and ADH-CMD-MNPs are spherical in shape and monodispersed. However, before immobilization of ADH, CMD-MNPs had a more uniform size, which was, on average, 28 nm, as investigated in and reported by our previous research^106^. After immobilization, it can be observed that CMD-MNPs are covered with a layer of enzyme due to epoxy activation, since such epoxy activation that is performed in a two-step mechanism allows ADH to covalently bind to CMD-MNPs. The sizes are slightly uneven and increased in diameter, ranging from 57 to 78 nm. Therefore, SEM images give additional confirmation that ADH has been successfully bound to the surface of CMD-MNPs by covalent attachment, resulting in the nano-sized product.

#### DLS analysis

The stability of the immobilized enzyme in the aqueous phase indicates an important influence on the potential for use, since most of reactions including enzymes are performed in the aqueous phase. Therefore, zeta potential was measured to indicate the stability of colloidal dispersion. The zeta potential of CMD-MNPs and immobilized ADH-CMD-MNPs was − 31.9 mV and − 26.8 mV, respectively (Fig. 5d). Both prepared CMD-MNPs and immobilized ADH-CMD-MNPs exhibit negative zeta potential, which indicates negatively charged hydroxyl and carboxyl groups of CMD present on the surface of nanoparticles. Zeta potential indicates that CMD-MNPs and ADH-CMD-MNPs show good dispersion in the aqueous phase.

In addition, the hydrodynamic size of the nanoparticles was measured and showed slightly larger (> 100 nm) nanoparticles. The results are presented in Fig. 5e. Differences in the particle size measured with DLS and other techniques (SEM or TEM) can occur because particle size measured by the DLS method represents the size of water-containing particles, which means that DLS measures the hydrodynamic radius of the nanoparticles. Also, magnetic nanoparticles tend to aggregate in order to reduce their surface energy. Many studies report a difference in their size obtained by SEM and DLS^96,106,123,124^.

The size distribution of nanoparticles is expressed in a polydispersity index (PdI). The PdI of ADH-CMD-MNPs is slightly higher (0.53), which is due to the layer of ADH immobilized on the surface of CMD-MNPs, which can also be observed from SEM images, while CMD-MNPs have a PdI of 0.31. Because ADH-CMD-MNPs are slightly uniform in size, the PdI index increases, as well.

## Conclusions

CMD-MNPs were successfully activated with epoxy groups using the activating agent EClH for covalent immobilization of ADH via a two-step immobilization protocol onto epoxy-activated supports. This optimized immobilization protocol resulted in not only 90% of expressed activity of immobilized ADH, but also in very good thermal and storage stability, an outcome which gives high importance to this study. Results indicated that epoxy-activated CMD-MNPs are favourable for ADH immobilization, which offers an opportunity for use in various biological applications (in the production of chiral pharmaceutical intermediates or in the preparation of fine chemicals). It also offers the opportunity for sensitive and rapid determination of ethanol in biosensor technologies, based on selective ethanol-converting enzymes, such as ADH. In our future studies, we plan to further investigate the optimized biocatalyst, perform additional experiments regarding the operational nature of the biocatalyst and apply it in bioreactor research.

## Methods

### Materials

Iron (III) chloride hexahydrate (FeCl_3_∙6H_2_O), iron (II) chloride tetrahydrate (FeCl_2_∙4H_2_O) and Coomassie brilliant blue were obtained from Merck. Ammonium hydroxide was purchased from Chem-Lab, Belgium. CMD sodium salt, EClH (1-Chloro-2,3-epoxypropane), sodium pyrophosphate, sodium phosphate, ethanol, β-nicotinamide adenine dinucleotide (β-NAD) and ADH from *S. cerevisiae* were purchased from Sigma-Aldrich. All reagents in this work were of analytical purity and used without further purification. In all experiments, deionized water was used.

### Synthesis of CMD-MNPs

CMD-MNPs were synthesized with co-precipitation of ferrous (Fe^2+^) and ferric (Fe^3+^) ions in a molar ratio 1:2. CMD was dissolved in distilled water with a final concentration of 0.5 g/mL. The solution of ferrous and ferric ions was mechanically stirred under N_2_ atmosphere at a temperature of 85 °C, and the CMD solution was added to the mixture. The mixture was precipitated drop-wise with an ammonia solution (NH_4_OH) and mechanically stirred for 1 h at 85 °C under N_2_ atmosphere. The obtained black precipitate was washed three times with distilled water and twice with ethanol. The preparation and synthesis of our CMD-MNPs with its detailed characterization analysis has already been published and is described in detail as CMD3-MNPs in^106^.

### Immobilization of ADH onto CMD-MNPs via epoxy activation

**Figure 6:**
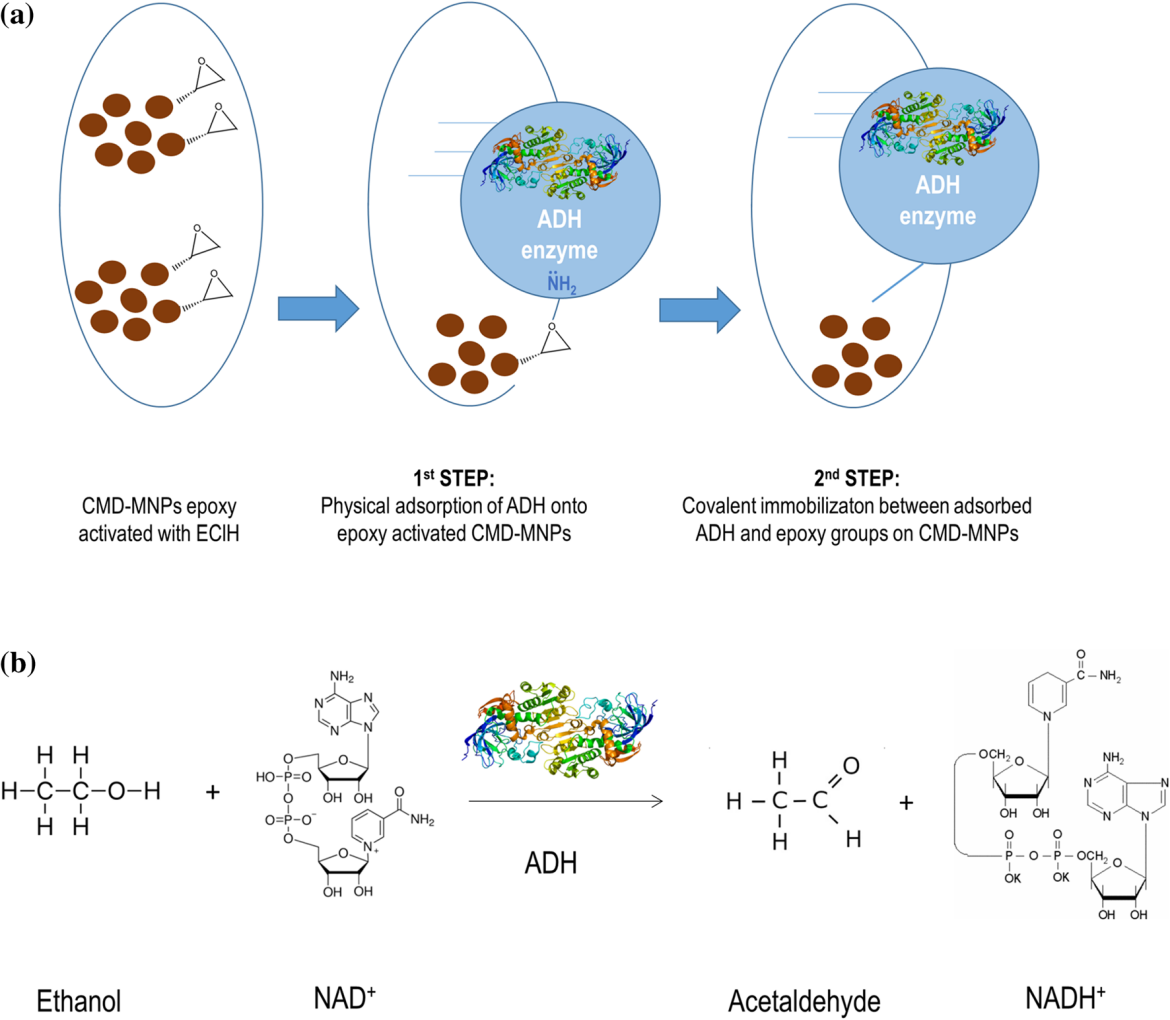
Schematic presentation of a two-step mechanism of immobilizing enzyme ADH onto epoxy activated CMD-MNPs (**a**) and reaction scheme of ethanol oxidation and acetaldehyde reduction catalyzed by enzyme ADH (**b**).

20 mg of CMD-MNPs were activated with 0.5 M EClH in the presence of 10 mM sodium phosphate buffer (pH 7.5). The CMD-MNPs were incubated with EClH at specific volumetric ratios at 300 rpm for 1 h at room temperature (20 °C). After 1 h, the supernatant was removed and 7.4 U/mL of ADH and sodium phosphate buffer were added to the CMD-MNPs at 300 rpm for 2 h at room temperature (20 °C) to obtain ADH-CMD-MNPs. After immobilization, the ADH-CMD-MNPs were washed twice with distilled water and magnetically separated using a permanent magnet. Subsequently, activity assay for ADH and protein determination using the Bradford method were performed to determine the expressed activity and immobilization yield of ADH-CMD-MNPs. Our immobilization protocol followed a two-step mechanism of immobilizing enzyme ADH onto epoxy activated CMD-MNPs. In the first step, the enzyme ADH was physically adsorbed to epoxy activated CMD-MNPs, in the second step, the enzyme ADH was covalently attached to the epoxy groups present on the surface of CMD-MNPs. The overall idea and a schematic presentation of our immobilization protocol is presented in Fig. 6a.

#### Assay for ADH activity

The activity of soluble and immobilized ADH was determined spectrophotometrically, using ethanol as a substrate and is based on the following reaction, presented in Fig. 6b. The standard reaction mixture in a total volume of 3 mL contained 22 mM sodium pyrophosphate, 3.2% (v/v) ethanol, 7.5 mM β-NAD, 0.3 mM sodium phosphate, 0.003 (w/v) % BSA and 0.075 units of ADH. The reaction was initiated by the addition of ethanol and β-NAD to the soluble or immobilized ADH, and subsequently, the increase in absorbance at 340 nm due to the formation of β-NADH was measured in an incubation time of 6 min.

The activity of soluble and immobilized ADH was calculated using the following equation:

1 $$ \frac{Units}{{mL}}enzyme = \frac{{((\Delta A _{340 nm} /\min ) \;of\; SAMPLE - (\Delta A_{340 nm} /\min )\;of\; BLANK) \times 3 \times df}}{6.22 \times 0.1} $$

where: 3—total volume (mL) of assay, df—dilution factor, 6.22—millimolar extinction coefficient of β-NADH at 340 nm, 0.1—volume (mL) of enzyme used.

Enzymatic activity of ADH was measured in triplicate by enzymatic assay for ADH by the Sigma-Aldrich protocol^125^, and the expressed activity was calculated from the following equation:

2 $$ {\text{Expressed}}\;{\text{ activity}}\; (\% ) = \frac{{{\text{activity}}\;{\text{ of }}\;{\text{immobilized}}\;{\text{ ADH}}}}{{{\text{ activity }}\;{\text{of }}\;{\text{soluble}}\;{\text{ ADH}}}} \times 100 $$

#### Protein determination and immobilization yield calculation

The protein amount of non-immobilized and immobilized ADH was determined by measuring the protein concentration by the Bradford method, using Bovine serum albumin (BSA) as a standard^126^. The amount of ADH immobilized on the surface of the CMD-MNPs was determined in the supernatant fraction (c_s_) and later calculated by subtracting the measured amount (c_s_) from the amount of non-immobilized ADH (c_e_).

Immobilization yield was calculated using the following equation:

3 $$ {\text{Immobilization }}\;{\text{yield}}\; (\% ) = \frac{{c_{e} - c_{s} }}{{c_{e} }} \times 100 $$

where: *c*_s_—protein concentration in supernatant fraction of immobilized ADH, *c*_e_—protein concentration of soluble ADH.

100% immobilization yield corresponds to the amount of immobilized enzyme of 0.1414 mg/mL.

All measurements for activity and protein determination were performed in triplicate and exhibited a standard deviation of less than 2%.

#### Effects of process parameters on ADH immobilization

Single-factor experiments were performed in order to study different immobilization conditions influencing the immobilization of ADH onto CMD-MNPs. Activating agent (EClH) concentration (2, 4, 6, 8% (v/v)), immobilization temperature (4 °C, 20 °C, 30 °C, 40 °C), rotation speed of immobilization process (200 rpm, 400 rpm, 500 rpm), pH of immobilization medium (5, 7, 7.5, 8, 9), time of immobilization (2 h, 3 h, 6 h, 12 h, 24 h) and enzyme concentrations (0.02 mg/mL, 0.03 mg/mL, 0.04 mg/mL) were applied to the immobilization process as variables. While optimizing each condition, previously optimized conditions always remained the same. Information on the constant process parameters during each optimization protocol is available in Table 2.

**Table 2:** Constant process conditions during the optimization process of each single-factor experiment.

Optimizing condition	Constant process conditions
EClH concentration [% (v/v)]	pH 7.5, 400 rpm, 4 °C, 0.02 mg/mL ADH, 2 h immobilization time
Immobilization temperature (°C)	4% (v/v) EClH, pH 7.5, 400 rpm, 4 °C, 0.02 mg/mL ADH, 2 h immobilization time
Rotation speed (rpm)	4% (v/v) EClH, pH 7.5, 4 °C, 0.02 mg/mL ADH, 2 h immobilization time
pH of the medium (/)	4% (v/v) EClH, 500 rpm, 4 °C, enzyme concentration 0.02 mg/mL, 2 h immobilization time
Immobilization time (h)	4% (v/v) EClH, pH 7.5, 500 rpm, 4 °C, enzyme concentration 0.02 mg/mL
ADH concentration (mg/mL)	4% (v/v) EClH, pH 7.5, 500 rpm, 4 °C, 2 h immobilization time

### Properties of immobilized ADH-CMD-MNPs

#### Thermal stability

Thermal stability of immobilized ADH-CMD-MNPs was studied by measuring expressed activity of the immobilized ADH-CMD-MNPs after incubation at two different temperatures, 20 °C and 40 °C after 3 h, 5 h and 24 h in relation to expressed activity of immobilized ADH-CMD-MNPs before incubation. The calculation followed Eq. (4):

4 $$ {\text{Expressed}}\;{\text{ activity }}\;(\% ) = \frac{{{\text{activity }}\;{\text{of }}\;{\text{immobilized }}\;{\text{ADH}}_{{{\text{after}}\;{\text{ incubation}}}} }}{{{\text{activity}}\;{\text{ of }}\;{\text{immobilized }}\;{\text{ADH}}_{{{\text{before }}\;{\text{incubation}}}} }} \times 100 $$

#### Storage stability

Storage stability of immobilized ADH-CMD-MNPs was studied by measuring expressed activity of immobilized ADH-CMD-MNPs at 4 °C for 22 days, with time intervals of a few days. Expressed activity was calculated according Eq. (5):

5 $$ {\text{Expressed}}\;{\text{ activity}}\; (\% ) = \frac{{{\text{activity }}\;{\text{of}}\;{\text{ immobilized }}\;{\text{ADH}}_{{{\text{after }}\;{\text{storage}}}} }}{{{\text{activity}}\;{\text{ of }}\;{\text{immobilized }}\;{\text{ADH}}_{{{\text{before }}\;{\text{storage}}}} }} \times 100 $$

#### Reusability assay

The reusability assay of immobilized ADH was performed in triplicate by activity measurement at room temperature at time intervals of 30 min. After each activity assay, the immobilized assay was washed with 1 mL of 10 mM sodium phosphate buffer (pH 7.5) and magnetically separated. After 30 min, the next activity measurement was carried out and compared to the first run (activity defined as 100%).

#### Determination of *K*_M_ and *v*_max_ for soluble and immobilized ADH

The enzyme kinetics parameters, Michaelis–Menten constant (*K*_M_) and maximum reaction velocity (*v*_max_) are characteristic kinetic constants that are used to evaluate the performance of immobilized enzymes. Therefore, the kinetic parameters of soluble and immobilized ADH were determined. *K*_M_ can be calculated using the Lineweaver–Burk diagram under optimum conditions^127^, following Eq. (6):

6 $$ \frac{1}{v} = \frac{{K_{M} }}{{v_{max} [S] }} + \frac{1}{{v_{max} }} $$

where: *v*—initial reaction velocity, *v*_max_—maximum reaction velocity, [*S*]—substrate concentration, *K*_M_—Michaelis–Menten constant.

Six different concentrations of the substrate ethanol were selected (0.3 M, 0.4 M, 0.5 M, 0.6 M, 0.7 M and 0.8 M) and investigated under optimal condition processes, obtained during the research: 4% (v/v) EClH, pH 7.5, 4 °C, 500 rpm, 2 h of immobilization time and with 0.02 mg/mL of ADH. Different concentrations were analysed, and the peak area of the product was used to express the initial reaction velocity (*v*). The *K*_M_ value of soluble and immobilized ADH was obtained by constructing the double reciprocal plot of 1/*v* versus 1/[*S*], based on Eq. (6).

### Characterization of ADH-CMD-MNPs

#### Fourier transform infrared spectroscopy (FT-IR)

FT-IR spectra were recorded to study the chemical bonds formed between ADH immobilized CMD-MNPs. FT-IR analysis of the samples was performed by pressing the samples to form a tablet, using KBr as the matrix. The spectra were detected over a range of 4000–500 cm^−1^ and recorded by a FT-IR spectrophotometer (Perkin Elmer 1600 Fourier transform infrared spectroscopy spectrophotometer).

#### Thermogravimetric analysis (TGA)

TGA was performed on a TGA/DSC1 (Schimadzu, IRAffinity-1, Mettler Toledo, Switzerland) under N_2_ atmosphere, with a heating rate of 10 °C/min from room temperature to 600 °C. Temperature precision was ± 0.3 °C and temperature accuracy was ± 0.5 °C.

#### Scanning electron microscopy (SEM)

The morphology and size of CMD-MNPs and ADH-CMD-MNPs was investigated by SEM analysis, using a scanning electron microscope (FE, SEM SIRION, 400 NC, FEI). The samples were measured on a gold (Au) substrate.

#### Dynamic light scattering (DLS)

The particle size distribution, hydrodynamic size and ζ-potential of the samples were measured using DLS (Zetasizer Nano ZS). Each diameter value was the average of three consecutive measurements. Higher values indicate a very broad size distribution, whereas lower values correspond to more or less monodisperse particle size distributions. Measurement was carried out under equilibrium conditions. The measured samples were dispersed in water with neutral pH at room temperature. Concentrations of all CMD-MNPs and ADH-CMD-MNPs were 2 mg/mL.

## Data Availability

All data generated or analysed during this study are included in this published article.
